# GC-MS studies of the chemical composition of two inedible mushrooms of the genus *Agaricus*

**DOI:** 10.1186/1752-153X-1-33

**Published:** 2007-12-20

**Authors:** Assya Petrova, Kalina Alipieva, Emanuela Kostadinova, Daniela Antonova, Maria Lacheva, Melania Gjosheva, Simeon Popov, Vassya Bankova

**Affiliations:** 1 Institute of Organic Chemistry with Centre of Phytochemistry, Bulgarian Academy of Sciences, 1113 Sofia, Bulgaria; 2 Department of Botany and Agrimeteorology, Agricultural University, 4000 Plovdiv, Bulgaria; 3 Institute of Botany, Bulgarian Academy of Sciences, 1113 Sofia, Bulgaria

## Abstract

**Background:**

Mushrooms in the genus *Agaricus *have worldwide distribution and include the economically important species *A. bisporus*. Some *Agaricus *species are inedible, including *A. placomyces *and *A. pseudopratensis*, which are similar in appearance to certain edible species, yet are known to possess unpleasant odours and induce gastrointestinal problems if consumed. We have studied the chemical composition of these mushrooms using GC-MS.

**Results:**

Our GC-MS studies on the volatile fractions and butanol extracts resulted in the identification of 44 and 34 compounds for *A. placomyces *and *A. pseudopratensis*, respectively, including fatty acids and their esters, amino acids, and sugar alcohols. The most abundant constituent in the volatiles and butanol were phenol and urea respectively. We also identified the presence of ergosterol and two Δ^7^-sterols. In addition, 5α,8α-Epidioxi-24(ξ)-methylcholesta-6,22-diene-3β-ol was isolated for the first time from both mushrooms. Our study is therefore the first report on the chemical composition of these two species.

**Conclusion:**

The results obtained contribute to the knowledge of the chemical composition of mushrooms belonging to the *Agaricus *genus, and provide some explanation for the reported mild toxicity of *A. placomyces *and *A. pseudopratensis*, a phenonomenon that can be explained by a high phenol content, similar to that found in other *Xanthodermatei *species.

## Background

Mushrooms in the genus *Agaricus *have a worldwide distribution, with up to 90 species recorded in Europe. The genus includes the most economically important and commercially cultivated mushroom in the world, *A. bisporus *(button mushroom) as well as many other edible species [1]. Some *Agaricus *species are inedible, including *A. placomyces *and *A. pseudopratensis *Bohus, which are similar in appearance to certain edible species, yet are known to possess unpleasant odours and result in gastrointestinal problems if consumed [2,3]. To the best of our knowledge, there is no information available in the literature concerning the chemical composition of these two species.

In this article, we report the results of the GC-MS analyses of volatile and polar compounds, in addition to the sterol fraction obtained from the fruiting bodies of *A. placomyces *and *A. pseudopratensis*. The results can help characterise the species investigated, indicate the presence of some biologically active compounds and shed light upon their reported mild toxicity.

## Results and discussion

The volatile fractions were obtained from the fresh mushrooms' fruiting bodies and analysed as described in the experimental section. The results obtained are outlined in Table 1. The most abundant constituent of the volatiles in both species was phenol: over 40% in *A. placomyces *and over 80% in *A. pseudopratensis*. Besides phenol, the main groups of compounds in the volatile fractions were fatty acids and their esters. Because the extraction was performed using ethanol, it is possible that the ethyl esters are artefacts. Our findings offer the first analytical proof of the presence of phenol in these two species.

**Table 1:** Volatile constituents of *A. placomyces *and *A. pseudopratensis *(% of the total ion current, GC-MS)*

**Compound**	***A. placomyses *(%)**	***A. pseudopratensis *(%)**
***Alcohols and phenols***		
Phenol	41.5	81
Benzyl alcohol	-	tr
***Aldehydes***		
Hexanal	0.9	-
2-decenal (90)	0.2	-
2,4-decadienal	<0.1	-
Undecenal (95)	0.5	-
***Ketones***		
2-undecanone	0.2	-
***Acids***		
Acetic acid	1.7	-
Hydroxy acetic acid	0.3	-
Pentadecanoic acid (93)	0.3	-
Hexadecanoic acid	4.5	5.5
Hexadecanoic acid – isomer	1.1	
9,12-Octadecadienoic acid (linoleic acid)	2.2	9.0
9-Octadecanoic acid (oleic acid)	3.7	-
***Ethers***		
Hydroquinone monopropyl ether (95)	3.3	-
***Estres***		
Acetic acid phenyl ester	0.5	-
Tetradecanoic acid ethyl ester	0.2	-
Hexadecanoic acid ethyl ester	5.1	8.4
Octadecanoic acid ethyl ester 18:0 (98)	0.5	1.2
Octadecenoic acid ethyl ester 18:1	1.1	-
9,12-Octadecadienoic acid ethyl ester (89)	10.1	-
***Terpenoids***		
Squalene	0.4	-

*The ion current generated depends on the characteristics of the compound concerned and is not a true quantification.

** In parenthesis: extent of matching (as a percentage) of MS data with the literature values, where the matching is not 100%

The phenol levels detected are not hugely surprising, given the numerous reports of the mushrooms' phenol-like odour, in addition to their belonging to the *Agaricus *section, *Xanthodermatei*, which is characterised by this typical unpleasant odour. It has been suggested that the production of phenol originates from an evolutionary ancestral biochemical shift, also demonstrated by the *Agaricus *species of the section *Xanthodermatei *in defence mechanisms [4]. According to Del Signore *et al*. [5] phenolic substances in mushrooms may be involved in a chemical defence mechanism against insects and micro-organisms, very similar to those described for certain plants (e.g. Salicaceae). Recently, it has been suggested that species with higher evolutionary positions in the section *Xanthodermatei *demonstrate higher phenol contents [4]. For this reason it could therefore be surmised that *A. pseudopratensis *is a more recently differentiated species than *A. placomyces*. However, this is only a preliminary hypothesis that will require further more detailed study.

The polar fractions obtained from the fungal species investigated were analysed by GC-MS after silylation. The derivatisation was necessary in order to increase the volatility of the polar fraction, thereby enabling its analysis by gas chromatography. The results obtained are outlined in Table 2. The presence of phenolics is clearly evident, as is the higher amount of phenol observed in *A. pseudopratensis*. This finding supports the suggestion of the defensive role of phenolics in section *Xanthodermatei *[6]. Another characteristic was the presence of the aromatic amino acids tryptophan and tyrosin, but only in *A. pseudopratensis*.

**Table 2:** Constituents of the polar fraction of *A. placomyces *and *A. pseudopratensis *(% of the total ion current, GC-MS)

Compound**	**Agaricus placomyses (%)**	**Agariricus pseudopratensis (%)**
***Alcohols and phenols***		
Ethylene diol	0.1	0.1
1,3-butane diol (96)	1.1	1.6
Phenol	0.6	1.0
Catechol	0.3	1.0
Resorcinol (98)	0.2	-
Hydroquinone	-	1.2
***Aminoacids***		
Alanine	1.5	1.3
Glycine (98)	0.2	0.2
Valine (96)	2.8	3.0
Leucine	1.5	2.7
Isoleucine(95)	0.5	1.8
Proline	0.3	0.9
Proline-5-oxo (90)	3.0	4.2
Threonine (95)	0.8	0.6
Phenylalanine	2.5	2.3
Tyrosine	-	1.4
Triptophan	-	1.4
***N – containing compounds***		
Urea	29.5	29.0
9-H-purine-6-OH (hypoxanthine)	0.4	-
6-amino-9-*β*-D-ribofuranosyl-9H-purine (91)	<0.1	-
Uracyl	-	0.1
Xanthine	-	0.1
***Acids***		
Butanedioic acid	0.5	1.1
Hydroxy butanedioic acid	1.6	2.6
2-butenedioic acid	0.7	0.6
Glutamic acid	0.4	-
Panthotenic acid	-	0.3
9,12-Octadecadienoic acid	-	0.1
Hexadecanoic acid	-	0.2
H_3_PO_4_	1.6	0.9
***Sugars and sugar alcohols***		
Glucitol (98)	35.0	10.0
Manitol (98)	-	9.8
Disaccharide	-	1.2

*The ion current generated depends on the characteristics of the compound concerned and is not a true quantification.

** In parenthesis: extent of matching (as a percentage) of MS data with the literature values, where the matching is not 100%

Sugar alcohols are amongst the major soluble carbohydrates found in fungi [7]. In the butanol fractions of both species we detected a sugar content of around 30%: glucitol in *A. placomyces *and a mixture of glucitol and mannitol in *A. pseudopratensis*. The most abundant constituent of the polar extracts was urea. This finding once again confirms that higher fungi of the family Agaricaceae accumulate substantial amounts of urea in their fruiting bodies [8]. It has been suggested that urea acts as an osmotically favourable nitrogen reserve for fungi [9].

We also investigated the sterol composition of the two *Agaricus *species. As expected the predominant sterol in *A. placomyces*, was ergosterol, although two others were detected, each possessing a Δ^7 ^double bond. In *A. pseudopratensis *only the latter two sterols were present in substantial quantities, with ergosterol detected in trace amounts.

Using column and preparative thin-layer chromatography, a crystalline compound was isolated from the extracts of both species and identified as 5α,8α-epidioxi-24(ξ)-methylcholesta-6,22-diene-3β-ol through comparison of its spectral data (MS, ^1^H- and ^13^C-NMR) with those reported in the literature [10]. The compound was first isolated from these species and might be regarded as an artefact produced from the oxidation of the corresponding Δ^5,7 ^sterol. It is noteworthy that this type of compound has recently been shown to express inhibitory behaviour against the HTLV-1 virus, in addition to demonstrating cytotoxic activity against human breast cancer cell line (MCF_7_WT) [10].

## Conclusion

The results obtained add to the knowledge on the chemical composition of mushrooms belonging to the genus *Agaricus*, and help provide further explanation for their reported mild toxicity, which we attribute to their high phenol content similar to that of other *Xanthodermatei *species [11].

## Experimental

### Collection of the samples

The macromycetes (age 3 – 5 days), were collected near Plovdiv, Bulgaria, in October 2005.

### Extraction

The fresh fruiting bodies of macromycetes (*A. pseudopratensis *– 105 g; *A. placomyces *– 245 g) were cut into small pieces and consecutively extracted with ethanol, ethanol-chloroform (1:1) and chloroform. The extracts were combined, water was added and the chloroform layers were removed and evaporated *in vacuo *to yield 1.88 g of dry residue for *A. placomyces *and 0.6 g for *A. pseudopratensis*.

### Isolation and analysis of volatile compounds

A portion of the chloroform extract (about 300 mg) was subjected to a four-hour distillation-extraction on Lickens-Nickerson apparatus [12]. The volatile compounds were extracted from the distillate with diethyl ether (yields of volatiles in Table 1), and analysed by GC/MS on a GC Hewlett Packard 6890 + MS 5973 (Hewlett Packard, Palo Alto, California, USA), with a HP5-MS capillary column (30 m × 0.25 mm, 0.25 μm film thickness, Agilent Technologies, Wilmington, Delaware, USA). The ion source was set at 250°C and the ionisation voltage at 70 eV. The temperature was programmed from 40 – 280°C at a rate of 6°C min^-1^, and a helium carrier gas was used used.

### Analysis of polar compounds

A portion of the n-butanol extract (5 mg) was dissolved in 50 μL of dry pyridine, before the addition of 75 μL of bis-(trimethylsilyl)-trifluoroacetamide (BSTFA). The mixture was heated at 80°C for 30 min and analyzed by GC/MS. The silylated extract was investigated by GC/MS using the same instrument described above, with a capillary column HP-5 (23 m × 0.2 mm, 0.5 μL film thickness, Agilent Technologies, Wilmington, Delaware, USA). A helium carrier gas was used. The temperature programmed at 100 – 315°C at a rate of 5°C min^-1^, with a 10 min hold at 315°C.

### Identification of compounds

Identification was carried out by searching commercial library databases. Some components remained unidentified, however, owing to both a lack of authentic samples and library spectra of the corresponding compounds.

### Isolation and identification of sterols

The chloroform extracts was subjected to column chromatography on silica gel with an *n*-hexane – acetone gradient (30:1 – 5:1) to produce several fractions. The third set of fractions, after further purification by preparative TLC (silica gel G, *n*-hexane – acetone 10:1), yielded sterol mixtures: 23 mg from *A. placomyes *and 7.9 mg from *A. pseudopratensis*, which were analysed by GC-MS. A gas chromatograph (Hewlett Packard 5890) linked to a mass spectrometer (Hewlett Packard 5972) with a capillary column SPB-50 (30 m × 0.32 mm, 0.25 μm film thickness) was employed. A helium carrier gas was used with a temperature programme set at 270°C – 290°C at a rate of 4°C min^-1 ^with a 20 min hold. The ion source was set at 250°C with the ionisation voltage at 70 eV.

From the fourth set of preparative TLC fractions (silica gel, *n*-hexane – methyl ethyl ketone 10:1) obtained from both species 5α,8α-epidioxi-24(ξ)-methylcholesta-6,22-diene-3β-ol was isolated (10.4 mg from *A. pseudopratensis *and 81 mg from *A. placomyces*) and characterised through comparison of its EIMS, ^1^H- and ^13^C-NMR spectra with literature data [10].

## Authors' contributions

AP performed the extractions, obtained the volatiles and isolated sterol mixtures, as well as participated in the data analysis and interpretation. KA participated in the data analysis and interpretation. EK performed the isolation and structural identification of the epidioxysterols. DA performed the GC-MS analyses. ML and MG participated in the collection and identification of mushroom material. SP conceived the study and helped draft the manuscript. VB participated in the design and coordination of the study, worked on the data analysis and interpretation and helped draft the manuscript. All authors read and approved the final manuscript.
